# Diagnostic evaluation of a point-of-care test for culture and microbial susceptibility testing in canine dermatological infections in clinical practice

**DOI:** 10.14202/vetworld.2020.521-529

**Published:** 2020-03-20

**Authors:** Roberta Perego, Eva Spada, Piera Anna Martino, Daniela Proverbio

**Affiliations:** Department of Veterinary Medicine (DIMEVET), University of Milan, via dell’Università 6, 26900 Lodi, Italy

**Keywords:** antimicrobial susceptibility test, dog, otitis externa, point-of-care test, superficial bacterial folliculitis

## Abstract

**Background and Aim:**

Empirical antimicrobial therapy is frequently given in superficial bacterial folliculitis (SBF) and otitis externa (OE) in dogs, especially for the initial clinical presentation. Culture and subsequent antimicrobial susceptibility testing (AST) are generally limited to chronic cases with poor response to initial therapy. Several factors contribute to the failure to implement the use of AST in veterinary practice, i.e., long laboratory turnaround time or special requirements for sample shipping. Point-of-care (PoC) testing might reduce laboratory turnaround time and costs and the risk of emergence of multidrug-resistant pathogens. This study evaluated the Speed Biogram™ PoC test in canine SBF and OE compared with conventional methods for culture and AST.

**Materials and Methods:**

Thirty-four canine samples were analyzed: eleven from SBF, seven from bacterial OE, four from mixed OE, six from *Malassezia* spp. OE, and six negative controls. Sensitivity (Se) and specificity (Sp) of the PoC test and the agreement between the PoC test and conventional methods were evaluated.

**Results:**

Se and Sp of PoC test in discriminating between healthy and unhealthy subjects were 100% (95% confidence interval [CI] 87.66-100.00) and 100% (95% CI 54.1-100.0), respectively. For bacterial identification, the k value was 0.532. Se and Sp of PoC tests for AST were 81.73% (95% CI 72.95-88.63) and 93.10% (95% CI 88.86-96.98), respectively with a total good agreement between tests (mean k=0.714), but major (8/27) and very major (19/27) errors were observed in 55% of bacterial conventional culture-positive samples.

**Conclusion:**

PoC test can identify dogs with SBF and OE, but AST is not sufficiently accurate. The lack of susceptibility testing for methicillin makes this test inappropriate for use in small animal practice.

## Introduction

Superficial bacterial folliculitis (SBF) and otitis externa (OE) are very common in dogs and typically require long-term treatment. SBF is an infection confined to the superficial portion of the hair follicle [1] and maybe secondary to local trauma, scratching, contamination due to poor grooming, seborrhea, parasitic infestation, hormonal factors, local irritants, or allergies [1]. The most frequent bacterial pathogen isolated from clinical canine samples [2] and the predominant agent of canine SBF [1] is *Staphylococcus pseudintermedius* (formerly *S. intermedius*). This is a commensal Gram-positive coagulase-positive bacterium that resides on the mucosal and skin surfaces of healthy dogs [2,3] and can be an opportunistic pathogen causing a skin infection. Infection results from an interaction between genetic, environmental, immunological, predisposing, and primary factors [2-5]. SBF is the primary reason for antimicrobial use in small animal practice [6]. Although less common, other Staphylococcus species, such as *Staphylococcus aureus* and *Staphylococcus schleiferi* [7] and other non-staphylococcal species [1] including *Escherichia coli*, *Proteus mirabilis*, *Corynebacterium* spp., *Bacillus* spp., *Pseudomonas* spp., and *Streptococcus canis* can colonize and infect canine skin [1,8], the clinical relevance of isolation of these species from SBF lesions is less clear [6]. OE is an inflammation of the external ear canal [1]. Bacteria are classified as secondary causes of OE, and common organisms isolated from affected dogs include S. pseudintermedius, *Proteus* spp., *Pseudomonas* spp., *Streptococcus* spp., *E. coli*, and *Klebsiella* spp. [1,9-12]. *Malassezia pachydermatis* is a common yeast that contributes to OE as a secondary cause [1]. *M. pachydermatis* is found in 62-76% of infected canine ears, frequently in combination with *Staphylococcus* spp. and may result in a superinfection after antibiotic therapy [13-15]. The diagnoses of SBF and OE are based on history, clinical signs, otoscopic examination in case of OE, and cytological examination of pustular exudate or auricular discharge [6,16]. The cytological examination requires a good optical microscope and an experienced operator.

Empirical antimicrobial therapy is frequently given in cases of SBF or bacterial OE [17]. Culture and subsequent antimicrobial susceptibility testing (AST) are generally reserved for chronic cases with poor response to initial therapy. This trend is unfortunate given the current concerns regarding antimicrobial use and emergence of multidrug-resistant (MDR) bacteria in animals, including companion animals [17]. According to the international guidelines for antimicrobial use in companion animal culture [18] and susceptibility testing should be performed on samples from small animals with suspected SBF or bacterial OE before treating with antibiotics [17]. Several factors contribute to the failure to implement this recommendation in veterinary practice [19], for example, long laboratory turnaround time, special requirements for sample shipping (i.e., transport medium), and the monetary costs for pet owners. In-house culture is a possible alternative to laboratory analysis, but the microbiological expertise required to accurately perform and interpret the diagnostic tests, as well as to perform routine quality control and manage the biohazard risks, is lacking in most in-clinic small diagnostic laboratories [17].

Point-of-care (PoC) testing for the bacteria and yeast involved in SBF and OE might reduce both turnaround time and costs for pathogen detection and AST of infections. Increased AST may reduce the risk of emergence of MDR pathogens. As demonstrated in human medicine, implementation of antimicrobial management at the clinic level has positive consequences on appropriate antimicrobial use, control of antimicrobial resistance and patient care [20]. A limited number of commercial PoC tests are available for on-site AST in veterinary clinics. Speed Biogram^™^ (Bio Veto Test, La Seyne sur Mer, France) is a PoC diagnostic test for small animals that simultaneously identifies bacteria and/or yeast on samples from skin and ears and the AST profile of the isolate (for bacteria only) through simple color changes at 18-48 h after inoculation.

The aims of this clinical study were: (i) To evaluate the performances of the PoC Speed Biogram^™^ for the detection and identification of yeast and bacteria in canine SBF and OE comparing the results with conventional laboratory culture; and (ii) to evaluate the PoC Speed Biogram™ for AST comparing the results with AST disk diffusion standard method.

## Materials and Methods

### Ethical approval and informed consent

All study procedures were performed in accordance with European legislation for animal research (2010/63/EU). Samplings were performed as part of the wellness assessment for apparently healthy dogs (control group) or routine diagnostic purposes (unhealthy dogs) under informed consent of the owners. Therefore, according to the Guidelines of our Institution, a formal approval from the Ethical Committee was not required since samples were taken with informed owner consent.

### Sample selection

Privately owned dogs (n=34) examined at the University of Milan with SBF, bacterial, mixed or *Malassezia* spp. bilateral OE and healthy subjects used as negative controls were included in the study.

For SBF and bacterial OE groups, dogs with compatible history, presence of clinical signs related to SBF [6] or OE [21] and the cytological identification (modified Wright’s rapid stain - Quick Panoptic Kit; Pokler Italia) of bacteria phagocytosed by neutrophil granulocytes [6,22] on cytological specimens from skin or ear were included in the study. For *Malassezia* spp. OE group, dogs with compatible history, clinical signs related to OE and the cytological identification (modified Wright’s rapid stain - Quick Panoptic Kit; Pokler Italia) of *Malassezia* spp. yeast with a median count >5 yeast per high power microscopical field (400×) on ear cytological specimens were included [22]. Those with both findings were included in the mixed OE group.

Control dogs were deemed healthy based on history, physical examination, and on the absence of neutrophil granulocytes, phagocytized bacteria and yeast <5 per high power microscopical field (400×) on ear and skin cytological specimens.

Dogs given local or systemic antibiotic/antimycotic drugs in the previous 2 weeks were excluded from the study. Antiseptic solutions were not used before sampling, and the investigator wore gloves for sample collection.

Informed owner consent was given for sterile single-use swabs (Gima SpA, Gessate, Milano, Italia) to be taken from dogs: (a) In case of SBF, two skin pustules on the same body region lanced with a 22G sterile needle and the pus collected on separate swabs; or (b) in case of OE, the external ear canal of right and left ear was swabbed at the junction between vertical and horizontal canals, for a total of two swabs for each ear. In healthy subjects, included as negative controls, two sterile swabs were taken from the right external ear canal at the junction between vertical and horizontal canals, and two sterile swabs were taken from the interdigital skin of a right forepaw.

One of the swabs was analyzed by the veterinary microbiology laboratory of the University of Milan for aerobic culture, and disk diffusion AST and the other immediately tested with Speed Biogram^™^. The following antibiotics, present in Speed Biogram^™^, have been tested with both methods (disk diffusion AST and Speed Biogram^™^): Amoxycillin (AMO), amoxycillin + clavulanic acid (AMC), cephalexin (CFL), doxycycline (DOX), enrofloxacin (ENR), marbofloxacin (MAR), difloxacin (DIF), clindamycin (CLI), gentamycin (GEN), and neomycin (NEO).

Treatment was given to the unhealthy dogs included in the study, as indicated by traditional culture and AST methods.

### Speed Biogram™ test

Briefly, for each test, the following kit was provided: (i) One disposable culture gallery, (ii) one disposable bottle of preservative medium (ingredients not provided by the manufacturer), (iii) one disposable bottle of culture medium (ingredients not provided by the manufacturer), and (iv) a Staph supplement bottle (ingredients not provided by the manufacturer) and a bottle of paraffin oil.

The culture gallery included as follows:

- One incubation limit (IL) well that changes color from red to orange/yellow if the incubation is valid and determines the time of control and antibiotic wells reading;
- Two control wells: A bacterial growth control well that changes color in the presence of bacterial concentrations >10^3^ CFU/mL and a negative control well that must remain colorless during the reading of the test.
- Six wells for the identification of pathogenic bacteria (bacteria with concentrations >10^3^ CFU/mL) (Tables-1 and 2) well for the identification of *Malassezia* spp. and *Candida* spp. yeasts.
- Thirteen antibiotics wells enabling the determination of AST of the pathogens present in the sample: AMO, AMC, CFL, DOX, ENR, MAR, DIF, FLU, CLI, SPI, GEN, NEO, and PXB. The method used to test the antimicrobial susceptibility is not reported by the manufacturer.

**Table-1:** Color changes for each bacterial well in case of positive identification (manufacturer’s instruction).

Name of well	Bacteria isolated	Initial well color	Final well color (positive identification)
STAPH	*Staphylococcus* spp.	Red	Yellow
STREP	*Streptococcus* spp.	Colorless	Grey-blue
ENTEROBACT	Enterobacteriaceae family	Red	Yellow/orange
PSEUDO	*Pseudomonas* spp.	Colorless	Navy blue
E. COLI	*Escherichia coli*	Colorless	Navy blue ring on the surface of well with contemporaneous change of color of ENTEROBACT well
PROTEUS	*Proteus* spp.	Yellow/orange	purplish pink with contemporaneous change of color of ENTEROBACT well

**Table-2:** Culture-positive samples with conventional culture and Speed Biogram™.

Origin	Conventional culture method	Speed Biogram™
OE	*Pseudomonas aeruginosa*	*Pseudomonas* spp.
OE	*Proteus mirabilis*	*Proteus* spp.,* Enterobacteriaceae*
OE	*Staphylococcus pseudintermedius*, *Proteus mirabilis*	*Staphylococcus* spp., *Proteus* spp.
OE	*Proteus mirabilis*, *Pseudomonas aeruginosa*	*Staphylococcus* spp.,* Proteus* spp., *Pseudomonas* spp., *Streptococcus* spp., *Enterobacteriaceae*
OE	*Staphylococcus pseudintermedius*, *Malassezia pachydermatis*	*Staphylococcus* spp., *Malassezia* spp.
SBF	*Staphylococcus pseudintermedius*, *Proteus mirabilis*	*Staphylococcus* spp.,* Proteus* spp., *Enterobacteriaceae*
SBF	*Pseudomonas aeruginosa*	*Pseudomonas* spp.
SBF	*Staphylococcus pseudintermedius*, *Pseudomonas aeruginosa*	*Staphylococcus* spp.,* Pseudomonas* spp., *Proteus* spp.
OE	*Staphylococcus pseudintermedius*, *Streptococcus* spp.	*Staphylococcus* spp.,* Streptococcus* spp.
SBF	*Staphylococcus pseudintermedius*, *Pseudomonas aeruginosa*, *Proteus mirabilis*	*Staphylococcus* spp.,* Proteus* spp., *Pseudomonas* spp., *Streptococcus* spp., *Enterobacteriaceae, Escherichia coli*
SBF	*Staphylococcus pseudintermedius*	*Staphylococcus* spp.
OE	*Streptococcus* spp.	*Streptococcus* spp.
SBF	*Staphylococcus pseudintermedius*	*Staphylococcus* spp.
OE	*Staphylococcus pseudintermedius*, *Malassezia pachydermatis*	*Staphylococcus* spp.,* Streptococcus* spp., *Malassezia* spp.
SBF	*Staphylococcus pseudintermedius*, *Pseudomonas aeruginosa*	*Staphylococcus* spp.,* Proteus* spp., *Pseudomonas* spp.
SBF	*Staphylococcus pseudintermedius*, *Proteus mirabilis*	*Staphylococcus* spp.,* Proteus* spp., *Pseudomonas* spp.
OE	*Proteus mirabilis*, *Malassezia pachydermatis*	*Proteus* spp., *Malassezia* spp., *Enterobacteriaceae*
SBF	*Staphylococcus pseudintermedius*	*Staphylococcus* spp.
SBF	*Staphylococcus pseudintermedius*, *Pseudomonas aeruginosa*, *Proteus mirabilis*	*Staphylococcus* spp., *Proteus* spp.,* Pseudomonas* spp.,* Streptococcus* spp.,* Enterobacteriaceae, Escherichia coli*
OE	*Staphylococcus pseudintermedius*, *Malassezia pachydermatis*	*Staphylococcus* spp.,* Streptococcus* spp.,* Malassezia* spp.
SBF	*Staphylococcus pseudintermedius*	*Staphylococcus* spp.
OE	*Proteus mirabilis*	*Proteus* spp.,* Enterobacteriaceae*
OE	*Malassezia pachydermatis*	*Malassezia* spp.
OE	*Malassezia pachydermatis*	*Malassezia* spp.
OE	*Malassezia pachydermatis*	*Malassezia* spp.
OE	*Malassezia pachydermatis*	*Malassezia* spp.
OE	*Malassezia pachydermatis*	*Malassezia* spp.
OE	*Malassezia pachydermatis*	*Malassezia* spp.

SBF=Superficial bacterial folliculitis, OE=Otitis externa

Immediately after sampling, the swab was placed in the bottle of preservative medium and vigorously shaken, pressed, and rotated against the walls of the bottle for few seconds to allow the material from the swab to transfer to the medium. After removing the swab, the bottle of the preservative medium was closed and shaken to mix the contents.

Using a disposable pipette, 3 drops of culture medium were transferred to the IL well. With a new disposable pipette, 4 drops of the seeded preservative medium were placed into the bottle of culture medium. The bottle of culture medium was closed and shaken.

Using another disposable pipette, 3 drops of the inoculated culture medium were distributed into each well of the gallery, with the exception of the IL well. In the *Staphylococcus* identification well (STAPH well), 2 drops of Staph supplement were added. Then 2 drops of paraffin oil were added to each well, with the exception of the wells identified as PSEUDO and *E. coli* wells.

Finally, the gallery was immediately incubated at +37°C in a dedicated incubator, not provided by the manufacturer.

**Figure-1:**
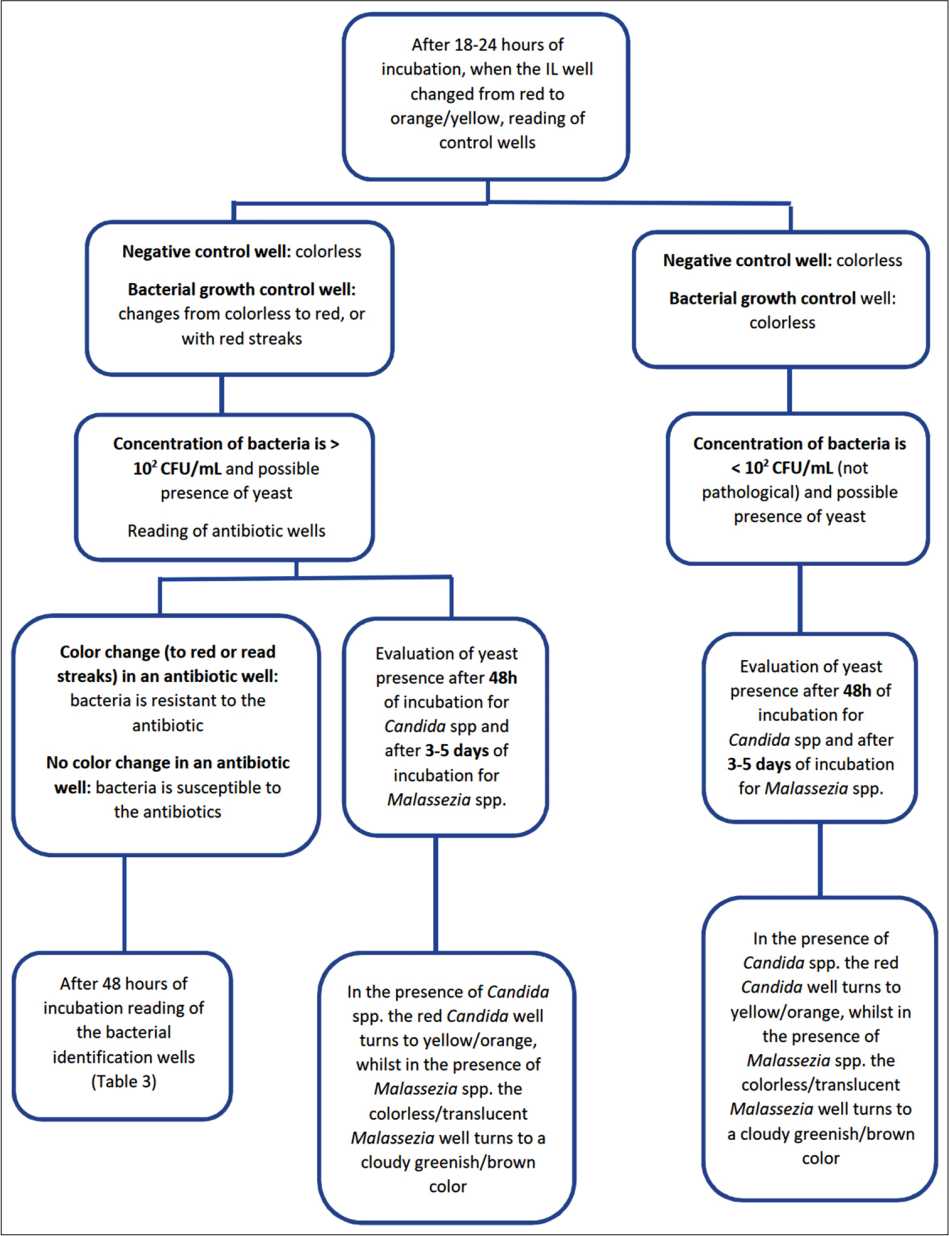
Algorithm for interpretation of Speed Biogram™ results.

The correct method of interpretation for Speed Biogram™ results is summarized in Figure-1.

**Figure-2:**
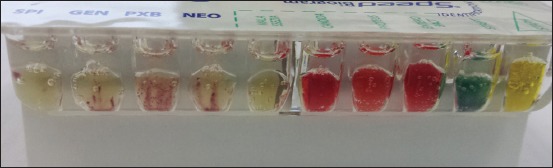
Detail of one of the sides of the gallery. On the left of the image is some antimicrobial susceptibility testing wells with red streaks (bacterial resistance), while on the right some wells for bacterial identification, with color change in the staphylococcus and streptococcus wells.

Antibiotic wells were interpreted only if the negative control well remained colorless, and red coloration was observed in the bacterial growth control well (Figure-2).

Each bacterial identification well was read assessing the color change (Figure-2). Table-1 shows the reported color changes for each bacterial well for different bacterial species – note multiple microorganisms may be present simultaneously.

As indicated by the manufacture if an identification well turned an intermediate color (often associated with turbid medium) after 48 h of incubation at +37°C (e.g., STAPH well turns from red to turbid orange), the well was re-examined 24 h later (at 72 h after inoculation), and bacterial identification was confirmed only if complete color change appeared. If the intermediate color persisted, the bacterial identification was considered negative.

To establish the repeatability of Speed Biogram^™^, one bacterial positive and one negative to standard culture method sample were analyzed 5 times on the same day.

Conservability was tested by performing the Speed Biogram^™^ test on two samples (negative and positive to standard culture method), inoculated in the preservative medium, cultured on the day of sampling and after 24 and 48 h of storage at +4°C, as indicated by the manufacturer.

### Conventional laboratory culture and AST methods

The strains used in this study were isolated as the unique or prevalent bacterial isolate from canine skin and/or external ear samples submitted for microbiological examination to Veterinary Microbiology Laboratory of University of Milan. Bacterial isolation was performed through cultivation on Tryptic Soy Agar plates containing 5% sheep blood (Microbiol, Cagliari, Italy) for 24 h at 37°C under aerobic conditions. Isolates were identified by morphology, hemolytic activity (i.e., for *Streptococcus*), Gram stain, catalase activity, and growth on mannitol salt agar selective medium and a commercial identification kit (API^®^ Staph, BioMériéux, France) was used for staphylococci identification; *Pseudomonas aeruginosa* was identified using Cetrimide agar (Thermo Fisher Diagnostics SpA., Milano, Italy) and *P. mirabilis* by observing typical swarming activity. The strains were tested for susceptibility to several antimicrobials (AMO, AMC, CFL, DOX, ENR, MAR, DIF, CLI, GEN, NEO, oxacillin, and others not present in Speed Biogram™) by the disk diffusion method (Kirby–Bauer tests) as follows. A 0.5 McFarland standardized suspension of each bacterial isolate was swabbed over the surface of a Mueller-Hinton agar plate (Thermo Fisher Diagnostics SpA., Milano, Italy) and paper disks (Thermo Fisher Diagnostics SpA., Milano, Italy) containing the different antibiotics (different µg per disk for each kind of molecules) were placed onto the inoculated surface. After overnight incubation at 37°C, the diameters of the zones produced by antimicrobial inhibition of bacterial growth were measured, and the result was interpreted as susceptible, intermediate, or resistant according to the criteria recommended by the Clinical and Laboratory Standards Institute [23]. Due to technical reasons flumequine, spiramycin, and PXB were not tested with standard method because the breakpoints were not recognized and available for disk diffusion.

For the isolation of yeasts, the samples were swabbed onto Sabouraud Dextrose Agar plates (Oxoid, Italy) and incubated for 72 h (or more until 5 days) at 25°C. The identification of *M. pachydermatis* was performed using Gram stain; the cells had a typical bottle shape.

### Statistical analysis

All statistical analyses were performed using commercial statistical software (MedCalc, v. 15.0.0, Mariakerke, Belgium). Descriptive statistics were used for demographic variables. Sensitivity (Se), specificity (Sp), negative predictive value (NPV), and positive predictive value (PPV) of the Speed Biogram^™^ for isolation and identification of bacteria and yeast were calculated using a 2×2 table based on the agreement with the culture conventional method. Se, Sp, NPV, and PPV for susceptibility testing were calculated by a 2×2 table using resistance (R) as a positive result and sensitivity (S) as a negative result for Speed Biogram^™^ relative to AST conventional method. Data on oxacillin and other antibiotics susceptibility obtained with conventional AST method but not with Speed Biogram^™^ were not considered. Data on flumequine, spiramycin, and polymyxin B susceptibility obtained with Speed Biogram™ but not with conventional AST method were not considered.

The agreement for the positive results between the Speed Biogram^™^ and the conventional culture and AST methods was evaluated using unweighted K statistic (k) with a 95% confidence interval (CI). The level of agreement was scored according to the following guidelines: 0: No better than chance; <0.20: Poor agreement; 0.21-0.40: Fair agreement; 0.41-0.60: Moderate agreement; 0.61-0.80: Good agreement; and 0.81-1.00: Very good agreement [24].

The presence of “major error” (ME) and “very major error” (VME) in the detection of antibiotic sensibility with Speed Biogram^™^ was also evaluated. A ”major error” occurs when the new test indicates resistance in a strain that is categorized as susceptible by the reference method (i.e., false negative). This error reduces the range of antimicrobial options available to the clinician and may lead to unnecessary use of broad-spectrum drugs, with potential negative consequences on the selection of resistance. A ”very major error” occurs when a strain categorized as resistant by the reference method is reported as susceptible by the test (i.e., false positive). This type of error has a greater impact on patient care, since the clinician may choose a drug that is unlikely to be effective against the strain causing infection, with all the negative consequences of treatment failure [25]. The acceptable inter method error rates of VME and ME are ≤1.5% and ≤3%, respectively [26].

## Results

A total of 34 canine samples from privately owned dogs were included and analyzed: Eleven samples (32%) from SBF, seven samples (21%) from bacterial OE, four samples (12%) from mixed OE, and six samples (18%) from *Malassezia* spp. OE was included, while three auricular and three skin samples (18%) were taken in six healthy dogs for use as negative controls. In mixed and *Malassezia* spp. OE, an average of 30 yeast per microscopic high power field (400×) was detected.

Using a conventional culture method, 18/34 (53%) samples were only bacterial culture-positive, 6/34 (18%) only *Malassezia* spp. culture-positive, 4/34 (12%) bacterial and *Malassezia* spp. culture-positive, and 6/34 (18%) bacterial and *Malassezia* spp. culture-negative. The same results were obtained with Speed Biogram^™^. The Se and Sp of Speed Biogram™ in discriminating between negative and positive samples were 100% (95% CI 87.66-100.00) and 100% (95% CI 54.07-100.00), respectively (NPV 100%, and PPV 100%). Unweighted k statistics demonstrated a k value of 1 (95% CI 1-1) with a very good agreement in the assessment of *Malassezia* spp. and bacterial infections between Speed Biogram™ and conventional culture method.

The conventional culture method of 22/34 bacterial positive samples resulted in the growth of *S. pseudintermedius* (n=15), *P. aeruginosa* (n=7), *P. mirabilis* (n=8), and *Streptococcus* spp. (n=2), alone or concurrent mixed infection.

Speed Biogram^™^ of the same 22/34 bacterial positive samples resulted in the growth of *Staphylococcus* spp. (n=16), *Pseudomonas* spp. (n=8), *Proteus* spp. (n=11), *Streptococcus* spp. (n=7), and *E. coli* (n=2), alone or concurrent mixed infection.

A lack of concordance between the two methods was found in eight bacterial positive samples in which Speed Biogram^™^ always not only detected the bacterium identified by the conventional culture method but also detected the presence of other associated bacteria (Table-2).

The Speed Biogram Enterobacteriaceae family well, which changes color in the presence of the genus *Proteus* spp. and/or *E. coli*, only reacted in 7/13 cases.

Unweighted k statistics demonstrated a k value of 0.532 (95% CI 0.319-0.745) for bacterial identification suggesting a moderate agreement between Speed Biogram™ and conventional culture method. Single or multiple false antimicrobial susceptibilities or resistances with Speed Biogram™ were seen in 12/22 (55%) bacterial culture-positive samples giving a total of 27 discrepancies. Specifically, in 19/27 cases (70%) Speed Biogram™ found susceptibility to antibiotics in bacteria that were shown to be resistant to that antibiotic with the conventional AST method (VME), while in the remaining 8/27 (30%) it showed antibiotic resistance in bacterial strains that were shown to be sensitive using the conventional AST method (ME) (Table-3). These errors occurred in different bacterial isolations and involved several categories of antibiotics. The sensitivity and specificity of Speed Biogram™ for susceptibility testing results were 81.73% (95% CI 72.95-88.63) and 93.10% (95% CI 88.86-96.98), respectively (NPV 85%, and PPV 91.4%). Unweighted k statistics demonstrated a good or very good agreement between Speed Biogram and conventional AST for tested antibiotics (average k=0.714) except for ENR, for which a moderate agreement (k=0.54 95% CI 0.184-0.892) was detected (Table-3).

**Table-3:** Antimicrobial susceptibilities or resistances with Speed Biogram™.

SAMPLE NUMBER	AMO	AMC	CFL	DOX	ENR	MAR	DIF	FLU	CLI	SPI	GEN	PXB	NEO
1.	R	R	R	R	R	R	R	R	R	R	**S**	S	**S**
2.	S	S	S	S	**S**	S	R	R	**S**	S	S	R	R
3.	**S**	**S**	S	R	S	**R**	S	R	R	R	S	R	R
4.	R	R	R	R	R	R	R	R	R	R	R	R	R
5.	S	S	S	S	S	S	S	S	S	S	S	S	S
6.	S	S	S	S	S	S	S	S	S	S	S	S	S
7.	S	S	S	S	**S**	S	R	R	**S**	S	S	R	R
8.	R	R	R	R	R	R	R	R	R	R	R	R	S
9.	S	S	R	S	R	R	R	R	R	R	R	R	R
10.	R	R	**S**	S	R	R	R	S	R	R	R	S	**S**
11.	S	S	S	**R**	S	S	S	R	R	R	S	R	R
12.	S	S	S	**R**	S	S	S	R	S	S	S	S	S
13.	**S**	**S**	S	S	S	**R**	R	R	R	R	R	R	R
14.	S	S	R	R	S	R	R	R	R	R	R	R	R
15.	R	R	R	R	R	R	R	R	R	R	**R**	R	S
16.	**S**	**S**	**S**	R	**S**	S	R	R	R	R	S	R	**S**
17.	S	S	S	S	S	S	S	S	S	S	S	S	S
18.	S	S	S	S	S	S	S	S	S	S	S	S	S
19.	R	R	R	S	R	R	R	R	R	R	R	S	R
20.	R	S	S	S	**R**	S	**R**	R	**R**	R	R	R	R
21.	S	S	S	S	S	S	S	S	S	S	S	S	S
22.	S	S	S	S	**S**	S	R	R	**S**	S	S	R	R
**K value**	**0.718**	**0.703**	**0.897**	**0.690**	**0.538**	**0.814**	**0.904**	**-**	**0.611**	**-**	**0.812**	**-**	**0.727**

With white background, the samples totally in agreement with conventional AST. With light grey background, the samples are not in agreement with conventional AST. In bold with dark grey background, the single false resistances (ME) and susceptibilities (VME). AMO=Amoxycillin, AMC=Amoxycillin + clavulanic acid, CFL=Cefalexin, DOX=Doxycycline, ENR=Enrofloxacin, MAR=Marbofloxacin, DIF=Difloxacin, FLU=Flumequine, CLI=Clindamycin, SPI=Spiramycin, GEN=Gentamicin, PXB=Polymyxin B, NEO=Neomycin, AST=Antimicrobial susceptibility testing, ME=Major error, VME=Very major error

The repeatability of the Speed Biogram™ was very good. The same results were recorded for culture and antimicrobial susceptibility in all five tests repeated on a singular positive and negative sample. The Speed Biogram™ also returned the same results in tests performed after 24 and 48 h storage periods.

## Discussion

Rational antimicrobial use is a key element for control of antimicrobial resistance, especially in veterinary dermatology. There is a European and global drive to reduce antimicrobial use in animals, including companion animals [6]. Selection of appropriate antimicrobials for treatment of SBF and OE is aided by rapid and reliable PoC tests to ensure that (i) antimicrobials are prescribed/used only when necessary, and (ii) the most appropriate drug is chosen based on antimicrobial resistance profiles [25].

To the best of our knowledge, this is the first attempt to determine the performance of a PoC test for yeast and bacteria identification and AST in veterinary dermatology. Our results show that Speed Biogram^™^ is easy to read and gives repeatable results. Our study also confirmed that the inoculated preservative medium remains stable for 48 h at +4°C, which is beneficial in clinical practice if sample processing is delayed. However, an incubator is required for the execution of Speed Biogram™ test, and the absence of this tool may limit the use of this PoC test in clinical settings.

Speed Biogram^™^ produces excellent results for the detection of bacterial and yeast infections in dogs. Its ability to discriminate healthy from pathological samples showed very good agreement with the conventional culture method, which is important to avoid the improper use of antibiotics in patients where there is no bacterial infection.

The disadvantage of Speed Biogram^™^ is the incubation time required before reading the result (48 h for bacteria and up to 72 h for *Malassezia* spp.). Although this is similar to that of conventional culture [27], it is much longer than the cytological examination, which requires only a stained slide prepared in a few minutes. The long incubation times of the Speed Biogram^™^ are a disadvantage, especially in the case of a simple *Malassezia* spp. OE, as the test does not give results until 2-3 days after inoculation and provides no details of the amount of yeast detected, or about drug resistance. However, cytological interpretation of skin or ear slide requires a trained operator and a good cytological technique [28], while the Speed Biogram™ results are very easy to interpret for first-time users with no other equipment (i.e., optical microscope).

In our study, the most commonly isolated bacteria were *Staphylococcus* spp., *Pseudomonas* spp., and *Proteus* spp., as in the previous literature data [1]. Although Speed Biogram™ identifies bacteria only to genus level, except for *E. coli*, this difference is not of great significance in clinical practice, where genus identification with appropriate AST may be sufficient to establish adequate antibiotic therapy. This is not true for some bacterial species, including *Staphylococcus* spp., because *S. aureus* and *S. pseudintermedius* have different susceptibility breakpoint with regard to the most important drug (oxacillin), not evaluated by Speed Biogram^™^. Speed Biogram^™^ does not allow determination of coagulase status of staphylococci and this could lead to the erroneous identification of coagulase-negative staphylococci, which normally have no pathogenic significance, such as *S. pseudintermedius*.

The moderate agreement with k statistics in bacterial identification between Speed Biogram^™^ and conventional culture is due to differences in eight bacterial positive samples in which Speed Biogram^™^ identified the bacteria cultured by the conventional culture method but in combination with other bacteria. The unreliability of these additional isolations is supported by the fact that in many identifications of *Proteus* spp. with the Speed Biogram^™^, the Enterobacteriaceae well, which should have been simultaneously colored, remained colorless. The presence of multiple identifications can be due to a poorly standardized inoculum and to the fact that the culture cannot be purified. Polymicrobial cultures are common in otitis and can occur from skin samples. In these cases, the relevance of the culture result and the selection of the isolate for AST need to be determined. The current recommendation for human wound infections is that the growth of potential pathogens should be reported, preferably semi-quantitatively and AST should be performed when a pathogen is isolated in pure culture or in abundance with minimal involvement of other micro-organisms [6]. Antimicrobial therapy should target microorganism with the greatest pathogenic potential. Indiscriminate reporting of AST profiles for microorganisms of minimal clinical relevance is discouraged to avoid unnecessary use of broad-spectrum antimicrobial drugs to cover the composite AST profiles of multiple isolates [17]. The polymicrobial inoculum highlighted with Speed Biogram^™^ may adversely affect susceptibility testing, which is based on the inoculation of the same culture medium.

Speed Biogram^™^ showed a mean good concordance on k statistics with conventional AST for nine tested antibiotics and moderate for ENR. This lesser concordance with ENR is not easily explained, since the antibiogram relative to other fluoroquinolones in the Speed Biogram^™^ yielded superior or almost optimal results, as in the case of DIF.

When analyzing individual samples, we found a 55% chance that the antibiogram of Speed Biogram™ is different from that suggested by conventional AST, and 70% of this is characterized by false susceptibility. These “major” or “very major” errors are extremely important – the Speed Biogram^™^ produced many “very major errors,” and therefore care must be taken in the interpretation of results as false antimicrobial susceptibilities may lead to inappropriate therapeutic choices, increasing the possibility of selection of MDR bacteria. Speed Biogram^™^ cannot test for the presence of methicillin-resistant *S. aureus* or methicillin-resistant *S. pseudintermedius*, as it lacks antibiotics such as oxacillin or cefoxitin [29]; further limiting its use, considering the importance of this data in public health and veterinary dermatology.

Our study has some limitations: It was not possible to provide data for flumequine, spiramycin, and polymyxin B – although these antibiotics were present in the Speed Biogram^™^, they are not available for disk diffusion AST or the breakpoints are not recognized. The absence of this data may have affected the statistical evaluation of the study. Another limit is the use of Kirby–Bauer disk diffusion test, based on serum level concentrations of drugs for AST for the treatment of OE, since topical therapy is typically used in most cases.

Furthermore, the variable sizes of the evaluation groups (dog with SBF, bacterial OE, *Malassezia* spp. OE, mixed OE, and healthy dogs) may have led to an overestimation of the sensitivity and specificity of Speed Biogram™ in the diagnosis of SBF and OE. In particular, ten dogs were enrolled with *Malassezia* spp. or mixed OE (with high cytological yeast numbers on microscopical high power field) and only six healthy dogs and, although Speed Biogram™ always correctly identified healthy and diseased subjects, its mechanism of identification is not clear, as the manufacturer does not provide a yeast growth control well with a detection limit for yeast isolation. Another possible limitation of our study is that dogs receiving antibiotic/antimycotic drugs in the previous 2 weeks were excluded. This exclusion criterion was implemented to avoid the administration of antibiotics as a confounding factor, but this choice led to the selection of a limited sample of subjects and did not test Speed Biogram^™^ in real clinical practice where it is necessary to perform sensitivity tests while the dog is receiving antibiotic or antifungal therapy.

## Conclusion

Speed Biogram^™^ is an easy to use PoC test for identifying dogs affected by SBF and OE. However, results of yeast identification cannot be interpreted for at least 48 h and precise identification of bacterial species responsible for the infection was not possible due to repeated, incorrect polymicrobial isolations.

Therefore, it can only be used in clinical practice in association with compatible clinical signs and cytological examination of samples. Speed Biogram^™^ may, however, be especially helpful in instances where cytological assessment from skin and auricular sites is not possible (inadequate equipment and training).

Speed Biogram^™^ cannot determine individual susceptibilities in polymicrobial isolations and reported several false antibiotic susceptibilities (VME). This limitation, along with the ability to identify bacteria only to the genus and the lack of susceptibility testing for methicillin, make Speed Biogram™ inadequate to guide antimicrobial therapy for SBF and OE in small animal practice and inappropriate for use in clinical dermatologic practice.

## Authors’ Contributions

RP, ES, and DP performed the literature search and sample collection. RP performed the Speed Biogram^™^. PAM performed conventional culture and susceptibility test methods. RP wrote the first draft of the article. All authors reviewed, read and approved the final version of the manuscript.
